# Phage lysin that specifically eliminates *Clostridium botulinum* Group I cells

**DOI:** 10.1038/s41598-020-78622-6

**Published:** 2020-12-09

**Authors:** Zhen Zhang, Meeri Lahti, François P. Douillard, Hannu Korkeala, Miia Lindström

**Affiliations:** Department of Food Hygiene and Environmental Health, Faculty of Veterinary Medicine, University of Helsinki, P. O. Box 66, 00014 Helsinki, Finland

**Keywords:** Food microbiology, Antimicrobials, Bacteriophages

## Abstract

*Clostridium botulinum* poses a serious threat to food safety and public health by producing potent neurotoxin during its vegetative growth and causing life-threatening neuroparalysis, botulism. While high temperature can be utilized to eliminate *C. botulinum* spores and the neurotoxin, non-thermal elimination of newly germinated *C. botulinum* cells before onset of toxin production could provide an alternative or additional factor controlling the risk of botulism in some applications. Here we introduce a putative phage lysin that specifically lyses vegetative *C. botulinum* Group I cells. This lysin, called CBO1751, efficiently kills cells of *C. botulinum* Group I strains at the concentration of 5 µM, but shows little or no lytic activity against *C. botulinum* Group II or III or other *Firmicutes* strains. CBO1751 is active at pH from 6.5 to 10.5. The lytic activity of CBO1751 is tolerant to NaCl (200 mM), but highly susceptible to divalent cations Ca^2+^ and Mg^2+^ (50 mM). CBO1751 readily and effectively eliminates *C. botulinum* during spore germination, an early stage preceding vegetative growth and neurotoxin production. This is the first report of an antimicrobial lysin against *C. botulinum*, presenting high potential for developing a novel antibotulinal agent for non-thermal applications in food and agricultural industries.

## Introduction

*Clostridium botulinum* is a Gram-positive, spore-forming anaerobic bacterium that produces botulinum neurotoxin (BoNT). BoNT causes botulism, a potentially fatal flaccid paralysis, in humans and animals^1^. The pathogenic process is initiated by spore germination and outgrowth of *C. botulinum* into vegetative, toxinogenic cultures in food or feed, or in the host gastrointestinal tract or in deep wounds^2^. Thus, botulism can manifest as an intoxication due to preformed BoNT or as a toxicoinfection from spores. *C. botulinum* is a heterogeneous taxon that comprises several genetically and physiologically distinct species with the common feature of botulinum neurotoxin production. Human botulism is predominantly associated with *C. botulinum* Group I and II. Sporadic cases of human botulism are also related to BoNT-producing *Clostridium baratii* (also called Group V) and BoNT-producing *Clostridium butyricum* (also called Group VI). Animal botulism is mainly associated with *C. botulinum* Group III. The link between botulism and *Clostridium argentinense* (also called Group IV) is unclear^2^. Botulism is a rare but extremely serious condition, with a single case activating a national alert and outbreak investigation, and causing enormous economic loss^3^. High-temperature processing in the food industry is the most effective method for inactivation of *C. botulinum* spores and the neurotoxin, but often compromises nutritional and sensory qualities of food products^4^. There is a growing interest in development of novel methods alternative to thermal process to control the botulism hazard^5,6^.

Bacteriophage lysins are natural hydrolytic enzymes that lyse host bacterial cells by disrupting the peptidoglycan layer^7^. In Gram-positive bacteria, many phage lysins consist of an amino-terminal catalytic domain, which cleaves one of the major bonds in the peptidoglycan, and a carboxyl-terminal binding domain, which recognizes unique carbohydrate epitopes of cell wall polysaccharides. The synergistic action of both domains confers potent antimicrobial activity selectively against specific host bacteria, which is a distinctive advantage over classical antibiotics and chemical preservatives. Moreover, the biodegradable nature of proteinaceous phage lysins is a considerable benefit for potential applications in food and feed safety and in therapeutics^8–10^. A number of phage lysins from a variety of host bacteria have been characterized^11,12^. Commercial applications of phage lysins have also progressed rapidly in developing treatments for microbial infections and solutions to improve food safety^13–15^. *Clostridium perfringens* is one of the most extensively studied anaerobic Gram-positive bacteria with characterized phage lysins and identified application potential. To date, ten lysins have been characterized^16–24^. A series of further studies have significantly improved the application potential of these lysins in prevention of *C. perfringens*-associated food-borne illness and enteric diseases of domestic animals, or as a diagnostic tool in the detection of *C. perfringens*^25,26^. Two phage lysins from *Clostridium difficile* and one from each of *Clostridium tyrobutyricum* and *Clostridium sporogenes* have been identified, all showing specific antimicrobial effects against the host bacterial species^27–30^. There is no report on phage lysins against *C. botulinum*. None of the currently known lysins was reported to possess lytic activity against *C. botulinum*. A phage lysin acting specifically against *C. botulinum* would allow a novel non-thermal solution to control botulism hazards in foods or feeds.

Here we report a putative phage lysin, CBO1751, identified in *C. botulinum* Group I strain ATCC3502. We demonstrate that recombinantly expressed CBO1751 has specific lytic activity against cells of *C. botulinum* Group I. We show that CBO1751 can effectively eliminate newly germinated cells, offering potential for early interruption of the pathogenic process of *C. botulinum*.

## Results

### Analysis of the putative phage lysin gene

**Figure 1:**
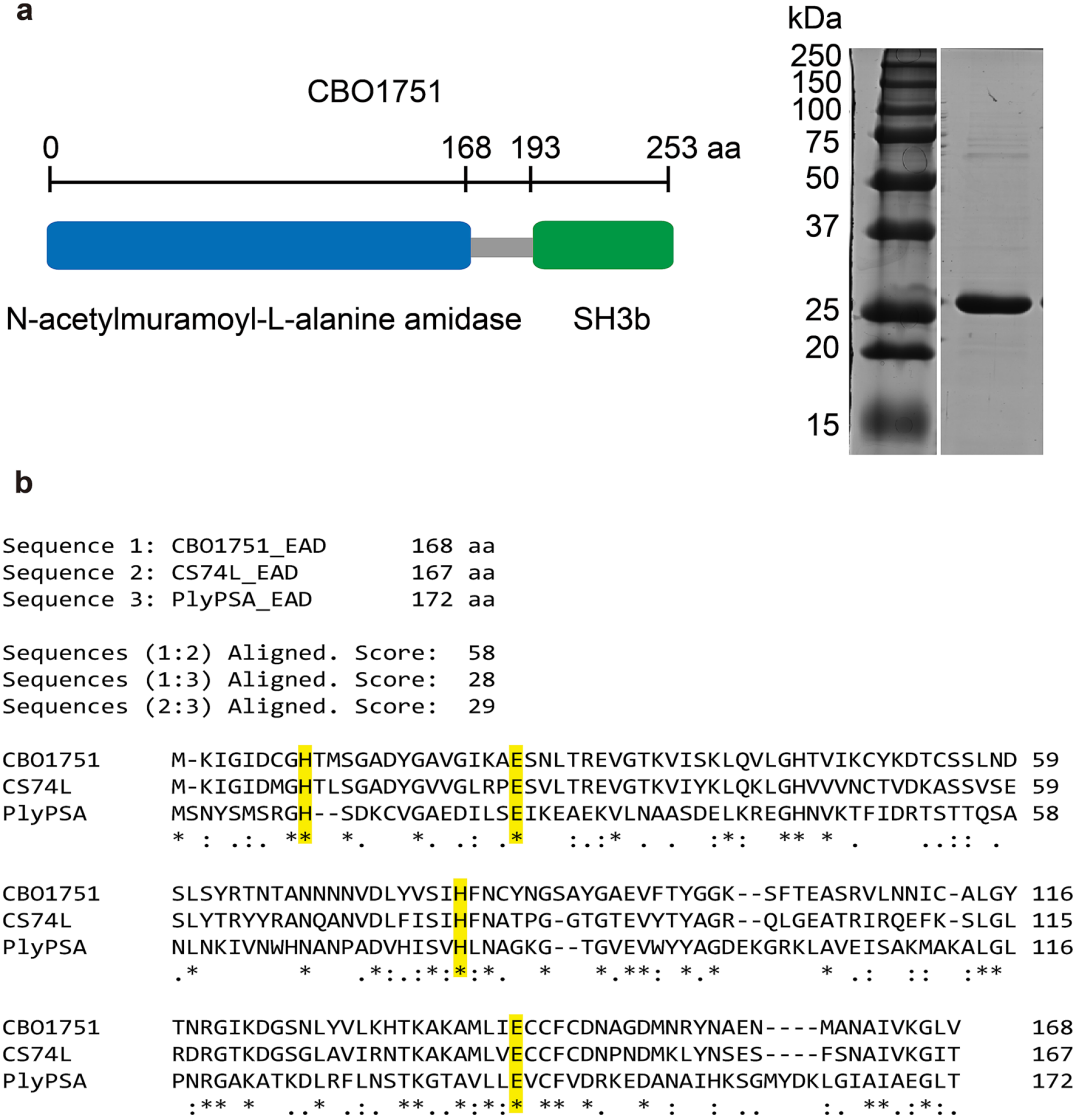
Features of CBO1751. (**a**) Schematic representation of the two domains of CBO1751 and SDS-PAGE analysis of purified CBO1751. Left lane of gel image, Precision Plus Protein Dual Color Standards (Bio-Rad, Hercules, USA). Right lane, eluted his-tagged CBO1751. The original gel is shown in Supplementary Fig. S2. (**b**) Amino acid sequence alignment of amidase EAD of CBO1751 with *Clostridium sporogenes* CS74L and *Listeria monocytogenes* PlyPSA. The conserved catalytic residues are marked in yellow.

CBO1751 was identified by prophage region analysis of the genome of *C. botulinum* Group I strain ATCC3502 using the PHASTER web server^31^. *cbo1751*, encoding a putative phage lysin, was found in one of two intact prophage regions. The amino acid sequence of CBO1751 is predicted to encode an enzymatically active domain (EAD) of *N*-acetylmuramoyl-L-alanine amidase (cd02696) on the *N*-terminal side and a cell wall‐binding domain (CBD) of bacterial Src homology 3 (SH3b) on the C-terminal side (Fig. 1a), suggesting that CBO1751 is an amidase lysin. CBO1751 shared an overall identity in amino acid sequence of 39% with the CS74L lysin of *C. sporogenes* and < 30% identity in amino acid sequence with other known lysins in *Clostridium* spp. (Supplementary Fig. S1). The amino acid sequences of EADs on the *N*-terminal side between the CBO1751 and CS74L lysins showed 58% identity (Fig. 1b), whereas the CBDs on the C-terminal side presented only 11% identity, suggesting that only the EADs of CBO1751 and CS74L are phylogenetically related. We further compared the amidase EADs of CBO1751 and CS74L with PlyPSA, a *Listeria* endolysin with four characterized catalytic residues responsible for zinc-dependent amidase activity^32^. Although the EADs of CBO1751 and CS74L shared < 30% amino acid identity to the EAD of PlyPSA, the alignment showed that all the corresponding catalytic residues are conserved in CS74L and CBO1751 (His9, Glu24, His80 and Glu141; Fig. 1b), implying that CBO1751 might have amidase activity similar to CS74L and PlyPSA. The SH3b CBD of CBO1751 showed high sequence divergence to the SH3b of other known lysins, including the well-studied SH3b domain (< 10% pairwise sequence identity) of staphylolytic lysins^33–35^.

We then investigated the prevalence of CBO1751 homologs in *C. botulinum* and in other bacteria. BLAST search found 45 CBO1751 homologs with > 80% amino acid identity in genomes of 152 *C. botulinum* Group I strains (Supplementary Table S1). BLAST search also identified 16 CBO1751 homologs with > 80% amino acid identity in genomes of 45 *C. sporogenes* strains. In addition, three CBO1751 homologs with > 80% amino acid identity were found in both *C. botulinum* Group I and *C. sporogenes* strains. BLAST analysis did not find any full-length CBO1751 homologs in *C. botulinum* Group II-IV or in other bacteria. The analysis indicates that CBO1751, a putative novel lysin, is highly conserved in a number of *C. botulinum* Group I and *C. sporogenes* strains.

### Characterization of lytic activity and host specificity of CBO1751

**Figure 2:**
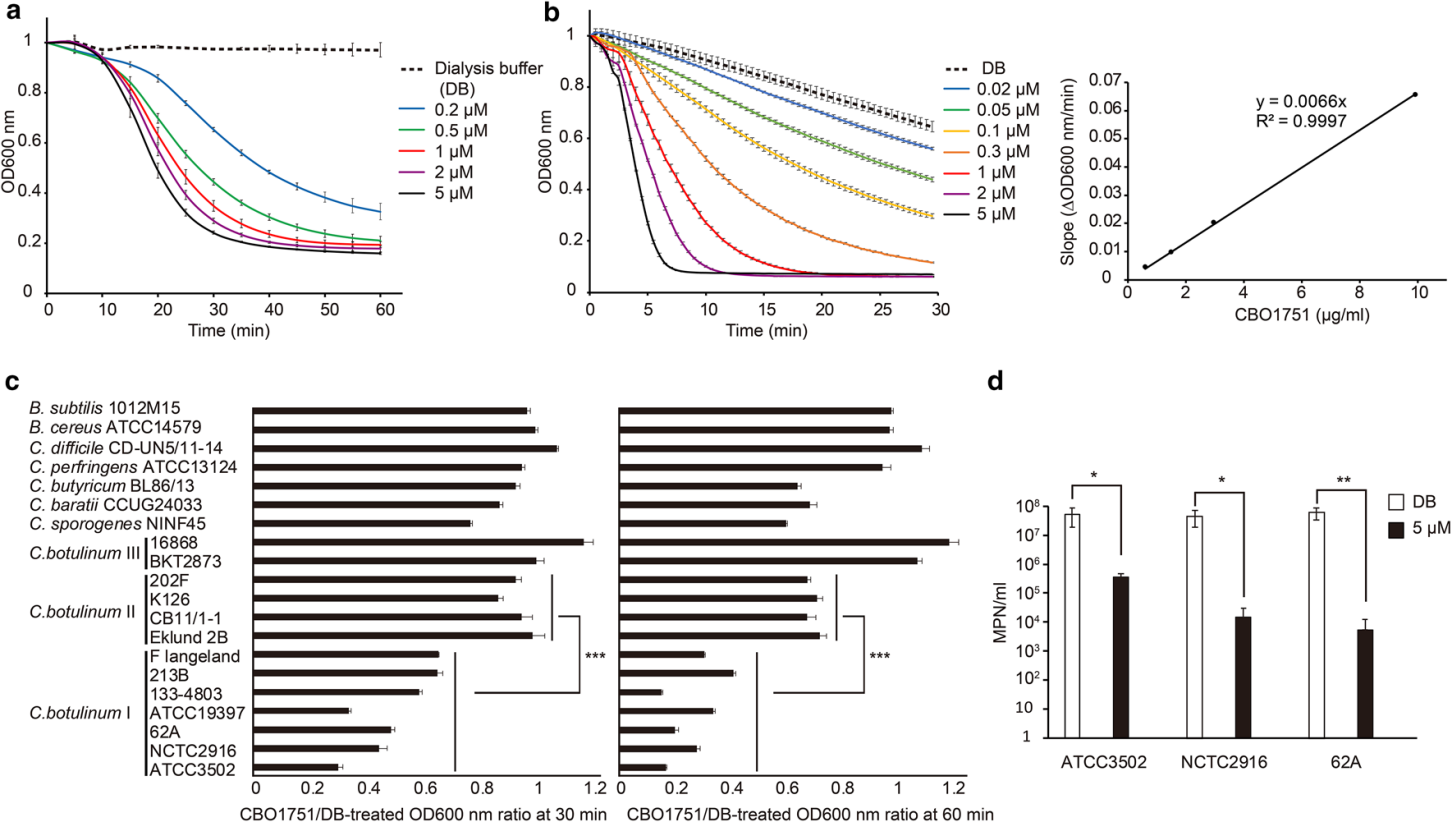
Lytic activity of CBO1751. (**a**) Lytic activity of CBO1751 as analyzed by measuring OD_600_ of *Clostridium botulinum* ATCC3502 vegetative cell suspension over 60 min after addition of DB or 0.2 to 5 µM of CBO1751. (**b**) Determination of lytic activity of CBO1751. Left: Lytic activity analysis by measuring OD_600_ of *C. botulinum* ATCC3502 frozen cell suspension over 30 min after addition of DB or 0.02 to 5 µM of CBO1751. Right: Linear regression plot of the slopes (ΔOD_600_/min) of lysis curves against the four CBO1751 concentrations (0.02, 0.05, 0.1 and 0.3 µM). All OD_600_ values of (**a**) and (**b**) were normalized to an initial OD_600_ of 1. (**c**) Lytic activity of CBO1751 against different *Firmicutes* strains as analyzed by comparing the ratio between OD_600_ values of 5 µM CBO1751-treated against DB-treated vegetative cell suspensions for 30 and 60 min. (**d**) Most probable number (MPN) enumeration of *C. botulinum* ATCC3502, NCTC2916 and 62A cells after treatment with 5 µM of CBO1751 or DB for 60 min. All results are presented as means of three replicates ± standard deviations. *, *P* < 0.05; **, *P* < 0.01; ***, *P* < 0.001.

Through cloning of *cbo1751* into pET21b vector and expression in *Escherichia coli* Rosetta 2(DE3) pLysS cells, we obtained the purified CBO1751 protein (yield approximately 15 mg/l of *E. coli* culture) with molecular weight of 29 kDa using affinity chromatography (Fig. 1a). To assess if CBO1751 exerts lytic activity on *C. botulinum*, turbidity reduction assay was performed using vegetative cell suspension of *C. botulinum* ATCC3502 with initial optical density at 600 nm (OD_600_) adjusted to approximately 1. In the cell suspension supplemented with protein-free dialysis buffer (DB), OD_600_ remained steady across all time points and was 0.94 for 60 min (Fig. 2a). However, a substantial reduction in OD_600_ was observed in cell suspensions supplemented with 0.2 to 5 µM of CBO1751, suggesting lytic activity of CBO1751 against *C. botulinum* ATCC3502 vegetative cells. The lytic activity of CBO1751 showed time and dose-dependency. The minimum OD_600_ (0.17) was reached at 60 min when treated with 5 µM of CBO1751 (Fig. 2a). To avoid variation in vegetative culture and cell suspension preparations between different batches, we prepared one stock of frozen cells of *C. botulinum* ATCC3502 and used this as substrate to determine the lytic activity of CBO1751. Unlike fresh vegetative cells, frozen cells with the control DB treatment showed marked drop of OD_600_, indicating autolysis during turbidity reduction assay (Fig. 2b). Nevertheless, CBO1751 still exhibited time and dose-dependent activity over a range of concentration from 0.02 to 5 µM. The OD_600_ values after subtraction of the control DB values were used to generate linear regression slopes (ΔOD_600_/min) of lysis curves. The enzymatic activity of CBO1751 was determined as 6,600 units/mg based on the linear equation of the slopes of the treatments of 0.02 to 0.3 µM CBO1751 (Fig. 2b).

We then tested the specificity of the lytic activity of CBO1751 against vegetative cell suspensions of a range of *C. botulinum* strains and other *Firmicutes* strains by comparing the CBO1751/DB-treated OD_600_ ratio at 30 min and 60 min. Of the seven *C. botulinum* Group I strains tested, the CBO1751/DB-treated OD_600_ ratio decreased markedly at both timepoints and varied from 0.18 to 0.41 at 60 min, whereas the values for four *C. botulinum* Group II strains were above 0.88 at 30 min and decreased only slightly to 0.68 to 0.72 at 60 min (*P* < 0.001, Fig. 2c). No marked reduction in OD_600_ was observed in *C. botulinum* Group III strains. These results suggest that CBO1751 has significant lytic activity against *C. botulinum* Group I strains, mild lytic activity against *C. botulinum* Group II strains, and no lytic activity against *C. botulinum* Group III strains. CBO1751 also showed a mild lytic activity against *C. sporogenes* NINF45, *C. baratii* CCUG24033 and *C. butyricum* BL86/13 with the CBO1751/DB-treated OD_600_ ratios at 60 min of 0.60, 0.69 and 0.64, respectively. No reduction in OD_600_ was observed for *C. perfringens* ATCC13124, *C. difficile* CD-UN5/11–14, *Bacillus cereus* ATCC14579 or for *Bacillus subtilis* 1012M15, suggesting that CBO1751 has no lytic activity against these *Firmicutes*.

We further performed viable cell enumeration of *C. botulinum* Group I strains ATCC3502, NCTC2916, and 62A cell suspensions treated with CBO1751 using the most-probable-number (MPN) method. Cell suspensions were equally distributed in two aliquots and treated with 5 µM of CBO1751 or the control DB for 60 min. To exclude the contribution of spontaneous cell death during the treatment, cell counts of the three strains after CBO1751 treatment were compared to those after DB treatment. While the cell counts of strains ATCC3502, NCTC2916, and 62A were estimated to be 5.5 × 10^7^, 4.7 × 10^7^ and 6.4 × 10^7^ MPN/ml after the 60-min DB treatment, counts of 3.7 × 10^5^, 1.6 × 10^4^ and 5.5 × 10^3^, respectively, were measured with the CBO1751 treatment (Fig. 2d), indicating about 2 to 4 log units difference in reduction of viable cell counts between CBO1751 treatment and the control DB treatment. The result provides further evidence for the lytic activity of CBO1751 against *C. botulinum* Group I strains.

### Effect of pH and salt on the lytic activity of CBO1751

**Figure 3:**
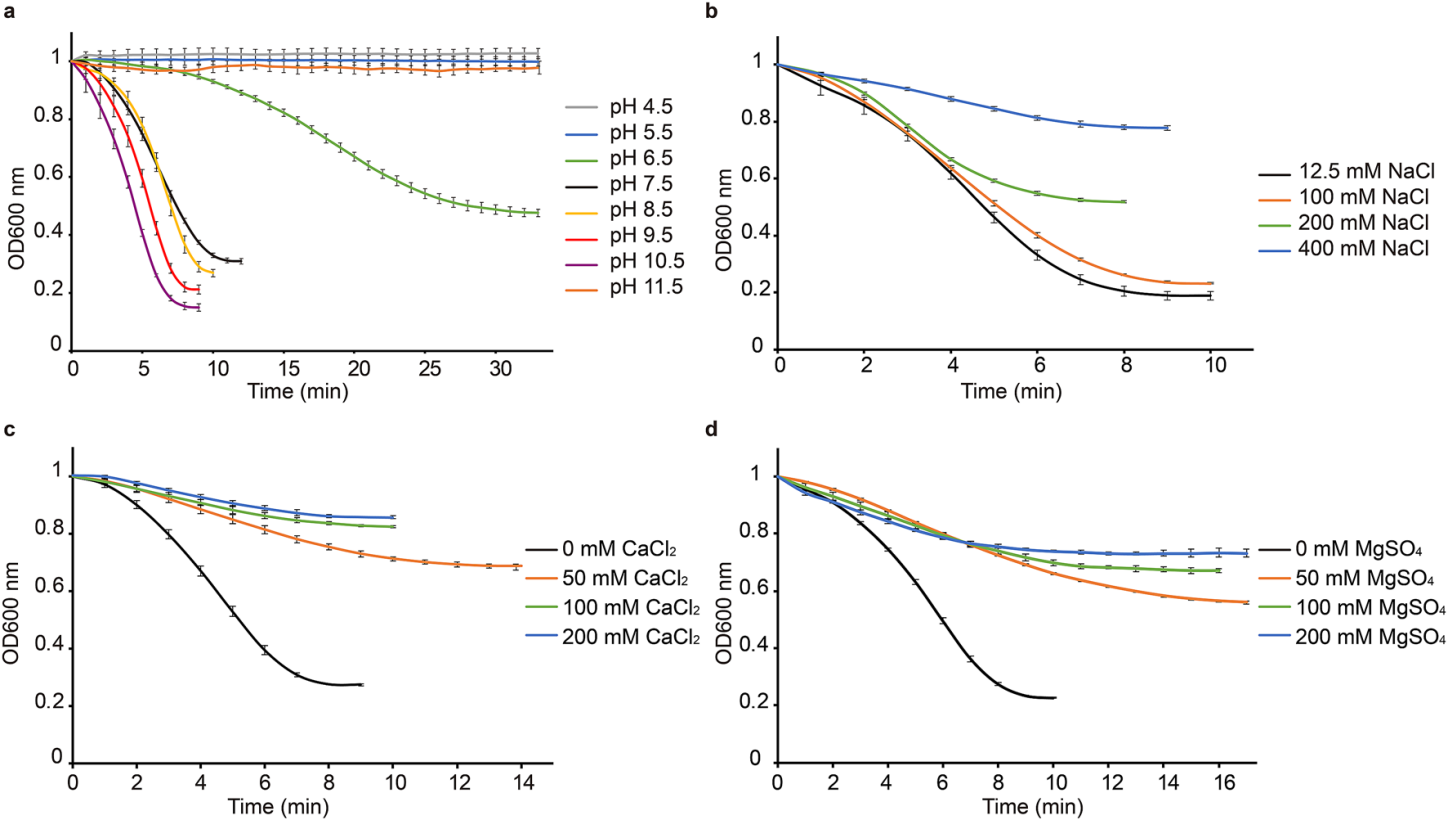
Effects of pH, NaCl, CaCl_2_ and MgSO_4_ on the lytic activity of CBO1751. Lytic activity analysis by measuring OD_600_ of *Clostridium botulinum* ATCC3502 frozen cells after addition of DB or 5 µM CBO1751 at pH from 4.5 to 11.5 (**a**) or in the presence of 12.5–400 mM NaCl (**b**), 0–200 mM CaCl_2_ (**c**) or 0–200 mM MgSO_4_ (**d**). All OD_600_ values were normalized to an initial OD_600_ of 1 and adjusted by subtraction of the corresponding DB control values. Results are indicated as means of three replicates ± standard deviations.

We conducted turbidity reduction assay to evaluate the influence of pH and salt type and concentration on the lytic activity of CBO1751 using frozen cells of *C. botulinum* ATCC3502. To exclude the effect of autolysis during the assays, the lysis curves with 5 µM CBO1751 treatment were presented after subtraction of the corresponding OD_600_ values of DB treatment under the same reaction conditions. CBO1751 at 5 µM caused a marked reduction of OD_600_ at pH from 7.5 to 10.5. The slopes of the lysis curves were similar at pH from 8.5 to 10.5 and were slightly deeper than that at pH 7.5, suggesting that an alkaline pH is more favorable for the lytic activity of CBO1751 than a neutral pH. The CBO1751 treatment caused only moderate reduction in OD_600_ at pH 6.5 and no reduction at pH 4.5, 5.5, and 11.5 (Fig. 3a). The results suggest that CBO1751 is active over a pH range of 6.5 to 10.5, with optimal activity at alkaline pH. We also evaluated the effect of salt ions on the enzymatic activity of CBO1751. Due to the addition of 5 µl of CBO1751 (dialyzed against DB buffer comprising 500 mM NaCl) to the final reaction mixture (200 µl), the lowest NaCl concentration tested was 12.5 mM. The CBO1751 treatment only caused a minor OD_600_ reduction in the presence of 400 mM NaCl (Fig. 3b). By contrast, similar lysis curves were observed with 12.5 and 100 mM NaCl. Due to cell autolysis triggered by the high concentration of NaCl, lysis curve at 200 mM NaCl flattened after 5 µM CBO1751 treatment for 5 min, but kept a deep slope during the first 4 min of CBO1751 treatment, which is comparable to that observed with 12.5 or 100 mM NaCl. These results suggest that NaCl up to 200 mM (1.2%) does not strongly affect the lytic activity of CBO1751. The lytic activity of CBO1751 was sensitive to the presence of divalent salts CaCl_2_ and MgSO_4_. CBO1751 treatment showed only a minor reduction of OD_600_ in the presence of 50 mM CaCl_2_ and MgSO_4_, and even less reduction with 100 and 200 mM CaCl_2_ and MgSO_4_ (Fig. 3c,d). The results suggest that a small amount of divalent cations significantly attenuates the lytic activity of CBO1751.

### Elimination of newly germinated cells by CBO1751

**Figure 4:**
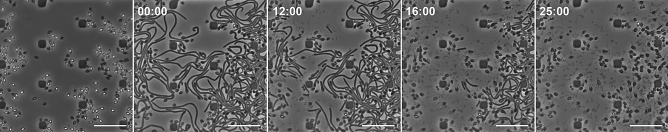
CBO1751 eliminates newly germinated cells of *Clostridium botulinum* ATCC3502 as analyzed by microscopy. First image from left, *C. botulinum* ATCC3502 spores in microfluidic chamber. Other images, representative time-lapse (min:sec) shots of cell lysing with 20 µM of CBO1751. Scale bars, 2 μm. Square-shaped dots are intrinsic pillars of the microfluidic chamber.

Since lysins kill bacterial cells by degrading the peptidoglycan cell wall, it is conceivable that CBO1751 may lyse newly germinated *C. botulinum* Group I cells once the peptidoglycan cell wall has been exposed. To confirm this, we prepared a germinating culture in a microfluidic system under anaerobic conditions and observed the effect of CBO1751 using time-lapse microscopy. After incubation of *C. botulinum* ATCC3502 spores in tryptone-peptone-glucose-yeast extract (TPGY) medium in a microfluidic chamber for 6 h, most of the phase-bright spores germinated and outgrew into thread-shaped vegetative cells that retained the empty shell of the spore coat at one end of the filament. Then, CBO1751 suspended in PBS (concentration optimized to 20 µM) was perfused into the chamber to replace TPGY. After CBO1751 perfusion for about 12 min, an intensive cell lysis was detected in newly germinated cells (Fig. 4 and Supplementary Video S1). Practically all cells were eliminated within 25 min. In contrast, germinated cells maintained their intact cell shape after perfusion of control DB suspended in PBS for 50 min, and no cell lysis was observed (Supplementary Fig. S3). These observations confirmed the lytic activity of CBO1751 against newly germinated *C. botulinum* Group I cells.

## Discussion

Bacteriophage lysins are the essential peptidoglycan-degrading proteins that are required by double-stranded DNA (dsDNA) phages for the lysis of host bacterial cell wall and release of their viral progenies to extracellular space. With dsDNA phages, lysins are exported by a holin-dependent or -independent mechanism through the bacterial cytoplasmic membrane into the periplasmic space and therefrom access the peptidoglycan substrate in the cell wall^36^. Therefore, phage lysins are primarily defined as endolysins. However, these enzymes can also degrade the peptidoglycan layer of Gram-positive bacterial cells exogenously and cause immediate cell lysis^37^. This feature underpins the broad application of phage lysins as natural antimicrobials with technical feasibility. In recent decades, the ever-increasing number of characterized phage lysins exhibits large antimicrobial potential against a list of pathogenic and food spoilage bacteria^11,12^.

Novel approaches alternative to thermal treatment would be a welcome addition in the toolbox for controlling the serious food and feed safety risks caused by the neurotoxigenic *C. botulinum*. Moreover, the use of natural antimicrobials in food products would reduce the need for chemical preservatives without compromising food safety. Here we demonstrate for the first time an active phage lysin efficiently killing *C. botulinum*. The lytic activity of CBO1751, originally identified in *C. botulinum* Group I strain ATCC3502, was verified with 5 µM CBO1751 leading to more than 2 log units reduction in *C. botulinum* cells in an hour. We also demonstrated efficient elimination of *C. botulinum* cells newly germinated from spores by addition of CBO1751, suggesting a great potential for CBO1751 as an effective control strategy to interrupt the pathogenic process of *C. botulinum* at an early stage.

pH is a major hurdle used in food preservation^38^. In general, pH < 4.6 is required to control *C. botulinum* spore germination and growth^39^. Food products and feeds with pH above 5 are favorable for *C. botulinum* growth^40^. Since some low-acid or non-acid foods can contain a relatively high prevalence of *C. botulinum* spores^41–43^, additional hurdles are needed to control the botulism hazard. Similar to phage lysins in other *Clostridium* spp.^27–30^, the lytic activity of CBO1751 exhibited a preference for neutral and alkaline pH, which allows CBO1751 to serve as an effective hurdle in these low-acid or non-acid foods to prevent production of neurotoxigenic cultures. Moreover, we observed robust cells lysis by CBO1751 in the presence of up to 200 mM (1.2%) NaCl, suggesting that the lytic activity of CBO1751 tolerates the NaCl levels added in many food products. While divalent metal cations were shown to be essential for the lytic activity of some zinc-dependent amidase lysins^44^, they exerted inhibitory effects on the lytic activity of some other amidase lysins^24,28^. Although CBO1751 has conserved zinc-binding active sites in the amidase EAD, we observed marked reduction of its lytic activity in the presence of 50 mM CaCl_2_ and MgSO_4_ (~ 0.6%). It is likely that an excess of divalent metal cations, once saturated the catalytic center of the amidase, affect the interaction between the lysin and the bacterial cell wall. The findings indicate that permitted levels of some divalent cation-based food additives might attenuate the effect of CBO1751 in elimination of *C. botulinum*. The proteinaceous nature of CBO1751 allows its safe application at high concentrations. Further studies are warranted to optimize the antibotulinal potency and synergistic effects of CBO1751 with other preservative hurdles in food products.

**Figure 5:**
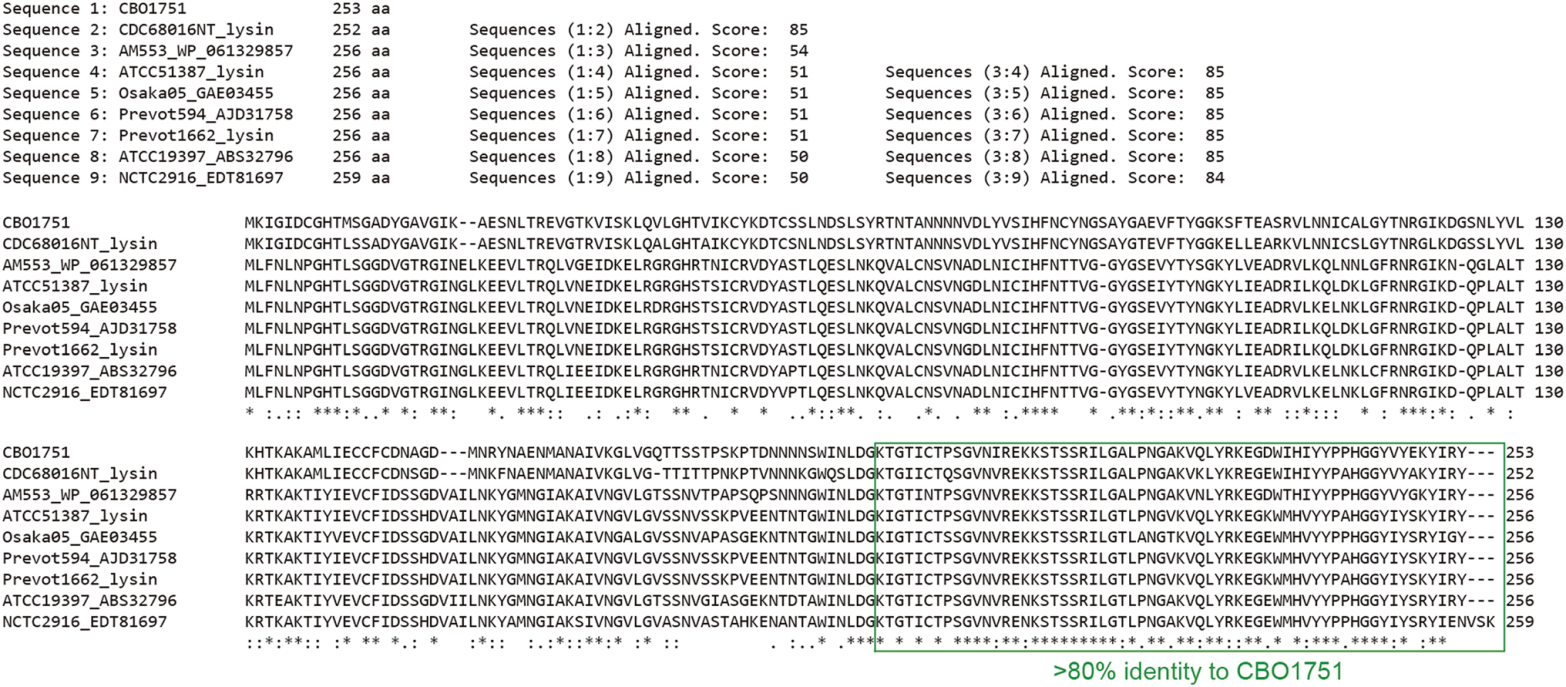
Amino acid sequence alignment of CBO1751 and putative lysins of *Clostridium botulinum* CDC68016NT (LAGL01000000, contig00983, nt 2654–3412), AM553 (WP_061329857), ATCC51387 (LAGD01000000, contig00030, nt 5143–5913), Osaka05 (GAE03455), Prevot594 (AJD31758), Prevot1662 (LAGM01000000, contig00035, nt 5087–5857), ATCC19397 (ABS32796) and NCTC2916 (EDT81697). Sequences encoding putative CBDs are indicated in a green box.

The host spectrum of CBO1751 is restricted to *C. botulinum* Group I, and to a lesser extent to *C. botulinum* Group II, *C. sporogenes, C. baratii* and *C. butyricum*. A rather rigid host specificity of CBO1751 was evidenced by a relatively high lytic activity of CBO1751 against all seven tested *C. botulinum* Group I strains, and a substantially lower activity against *C. botulinum* Group II, *C. sporogenes*, *C. baratii* and *C. butyricum* strains, and no activity against other *Firmicutes*. This is consistent with the identification of highly conserved CBO1751 homologs (> 80% amino acid identity) in many *C. botulinum* Group I genomes but not in any strain of Group II, *C. baratii* and *C. butyricum*, and other Firmicutes. *C. sporogenes* is considered a nontoxigenic variant of *C. botulinum* Group I and forms a distantly related clade in the Group I phylogeny. Recent studies suggest that the *C. sporogenes* clade also contains some botulinum neurotoxigenic strains including CDC68016NT, AM370, AM1195, AM553, ATCC51387, Osaka05, Prevot594 and Prevot1662^45,46^. CDC68016NT, AM370 and AM1195 carry putative phage lysins with > 80% amino acid identity to CBO1751 (Fig. 5 and Supplementary Table S1), implying that the three toxigenic *C. sporogenes*-like strains might be highly susceptible to CBO1751. However, strains AM553, ATCC51387, Osaka05, Prevot594 and Prevot1662 were found to possess putative lysins with only ~ 50% amino acid identity to CBO1751 (Fig. 5). Similarly, ATCC19397 and NCTC2916, two strains in the *C. botulinum* clade of Group I phylogeny, do not carry any highly conserved CBO1751 homolog, but possess CBO1751 homologs with 50% overall sequence identity and showed efficient lysis upon CBO1751 treatment. Intriguingly, the CBD of these CBO1751 homologs shared > 80% amino acid identity to the CBD of CBO1751 (Fig. 5). The CBD is generally responsible for host specificity of lysins by binding to unique carbohydrate epitopes of cell wall polysaccharides in select bacterial species. Therefore, CBO1751 seems to have a broad host spectrum encompassing both clades in the *C. botulinum* Group I phylogeny. Further tests with more Group I strains will offer a better understanding of the host specificity of CBO1751. Moreover, to combat the wide heterogeneity of all BoNT-producing species, it is important to characterize lysins that target specifically each phylogenetically distinct group. This will offer not only additional antibotulinal lysins, but also a set of active EADs and CBDs for further domain shuffling and engineering to enhance the lytic activity and broaden the host spectrum, ultimately providing an all-encompassing-tool to control the botulism hazard.

As an example of a lysin CBD, the SH3b domain is found in many bacteria and is proposed to recognize peptidoglycan stem peptides and cross-bridges of the bacterial cell wall^33–35^. In the case of the bacteriolysin lysostaphin, the SH3b domain specifically binds to pentaglycine cross-bridges that are a unique feature of staphylococci^33^. We found that the CBO1751 CBD was highly conserved (> 80% amino acid identity) in a large number of *C. botulinum* Group I strains, but not in *C. botulinum* Group II or III, or other BoNT-producing *Clostridium* species. Except for some putative amidases in *Clostridiaceae*, CBO1751 CBD shared < 50% amino acid identity to some hypothetical surface proteins or peptidases in the family *Bacillaceae.* We speculate that the CBD of CBO1751 might recognize the assumingly conserved peptidoglycan structure in *C. botulinum* Group I strains, thereby conferring its highest lytic activity against *C. botulinum* Group I. Further characterization of the CBO1751 CBD substrate will reveal the cell wall features of *C. botulinum* Group I. Due to specific binding, lysin CBDs show great potential for use as diagnostic tools in bacterial detection^13^. A variety of CBD-based methods were developed for rapid detection of *L. monocytogenes*^47^, *Staphylococcus aureus*^48^, *B. cereus*^25,49^, *C. perfringens*^25^, and *C. tyrobutyricum*^50^. The rigid host specificity of CBO1751 might favor the development of a CBO1751 CBD-based diagnostic tool for detection of *C. botulinum* Group I in food and clinical samples.

In summary, our data show that the putative lysin CBO1751 efficiently lyses *C. botulinum* Group I cells and may provide a useful tool in non-thermal control of *C. botulinum*. Further studies are required to develop CBO1751-based antibotulinal applications in food products and rapid detection methods for *C. botulinum*, both approaches providing valuable novel contributions to controlling the botulism hazard.

## Materials and methods

### Computational analysis of phage lysin

The genome of *C. botulinum* Group I strain ATCC3502 (Genbank accession number AM412317) was analyzed for prophage regions using the PHASTER web server. Sequence homology analysis was performed using BLASTp and NCBI Conserved Domain Search tool, and sequence alignment was done with ClustalW.

### Bacterial strains and culture conditions

The bacterial strains used in this study are listed in Supplementary Table S2. All clostridia were cultured in anaerobic TPGY medium at 30 °C (*C. botulinum* Group II strains) or 37 °C in an anaerobic workstation with an atmosphere of 85% N_2_, 10% CO_2_, and 5% H_2_ (MK III; Don Whitley Scientific Ltd., West Yorkshire, UK). *B. subtilis* 1012M15, *B. cereus* ATCC14579 and *E. coli* Rosetta 2(DE3) pLysS cells (Merck Millipore, Darmstadt, Germany) were grown in Luria–Bertani (LB) medium at 37 °C. When appropriate, growth media were supplemented with 100 μg/ml ampicillin and 34 μg/ml chloramphenicol.

### Expression and purification of recombinant CBO1751

CBO1751 (GenBank accession number CAL83288) was commercially synthesized and cloned in the NheI/SalI restriction sites of pET21b to generate a C-terminal fusion construct with 6 × His tag (GenScript Biotech, Leiden, Netherlands). The vectors were transformed into *E. coli* Rosetta 2(DE3) pLysS cells (Merck Millipore). The expression of His-tagged CBO1751 was induced with 1 mM IPTG at 30 °C for 8 h. The expressed protein was purified using metal-chelate affinity chromatography with Ni–IDA resin (Merck Millipore) as previously described^51^. Eluted proteins were examined by SDS-PAGE prior to dialysis (MWCO 8 kDa, Spectrum Labs, New Brunswick, NJ, USA) against 500 ml of dialysis buffer (DB: 500 mM NaCl, 50% glycerol, 20 mM Tris–HCl, pH 7.9) overnight at 4 °C. Protein concentrations were determined using the Pierce™ BCA Protein Assay Kit (Thermo Fisher Scientific Oy, Vantaa, Finland) with bovine serum albumin (Merck Millipore) as a standard.

### Lytic activity analysis

*Clostridium* strains were cultured to mid-exponential phase (OD_600_ 0.6–0.7) and cells were collected by centrifugation at 5000 × *g* for 5 min. Pellets were washed gently, resuspended in PBS buffer and adjusted to approximately OD_600_ of 1. A stock of purified CBO1751 was concentrated by Amicon Ultra-15 centrifugal filter (Merck Millipore) and adjusted by DB to the concentration of 200 µM for lytic activity analysis. Turbidity reduction assay was made by measuring the OD_600_ of the cell suspensions immediately after adding CBO1751 in a 96-well plate in a volume of 200 µl/well using Multiskan™ Ascent microplate reader (Thermo Fisher Scientific). For each reaction, 195 µl of cell suspension was mixed with 5 µl of DB-diluted CBO1751 to reach final concentrations of 0.2 to 5 µM, or with 5 µl DB as control and incubated at room temperature. The OD_600_ of the cell suspensions was measured every 5 min over 60 min. Cell counts were determined after treatment for 60 min using the MPN method as described^52^.

To determine the lytic activity, a frozen cell stock was prepared from *C. botulinum* ATCC3502 grown until OD_600_ 0.6–0.7. Cells were washed gently, resuspended in 20% glycerol and flash frozen in liquid nitrogen. Turbidity reduction assay was conducted as above except that OD_600_ was measured every 30 s over 30 min. Data analysis was performed as previously described^44,53^. OD_600_ values were normalized to an initial OD_600_ of 1 and adjusted by subtracting the corresponding DB control values [adjusted value = normalized value + (1 − normalized DB control value)]. The deepest slopes (*R*^2^ > 0.995) of lysis curves were selected for linear regression analysis to calculate lytic activity. The activity unit was defined as the amount of enzyme resulting in a reduction of 0.001 OD unit/min in the OD_600_ of *C. botulinum* ATCC3502 frozen cells.

To assess the effect of pH on the CBO1751 lysin activity, frozen *C. botulinum* ATCC3502 cells were equally suspended in universal buffers with pH ranging from 4.5 to 11.5 and adjusted to OD_600_ of approximately 1. The universal buffer was prepared by mixing 20 mM each of boric acid and phosphoric acid, followed by titration with sodium hydroxide^54^. For each reaction, 195 µl of cell suspension was mixed with 5 µl of DB-diluted CBO1751 (final concentration 5 µM) or with 5 µl DB as control. The final pH of each reaction was verified by pH meter. The turbidity reduction assay was carried out at room temperature with the measurement of OD_600_ every 1 min over 60 min. To evaluate the effect of salts on the CBO1751 activity, frozen *C. botulinum* ATCC3502 cells were equally distributed in 50 mM Tris–HCl buffer (pH 7.5) with NaCl (final concentration of 12.5, 100, 200 or 400 mM), CaCl_2_ (final concentration of 0, 50, 100 or 200 mM) or MgSO_4_ (final concentration of 0, 50, 100 or 200 mM) and adjusted to OD_600_ of approximately 1. Turbidity reduction assay was performed as above by adding 5 µl of DB-diluted CBO1751 (final concentration 5 µM) or 5 µl DB to cell suspensions. OD_600_ values were normalized and adjusted as described above.

All experiments were conducted with three biological replicates. Student's *t*-test was used for statistical comparisons.

### Time-lapse imaging

*C. botulinum* ATCC3502 spores were prepared as described^55^ and fixed into a microfluidic plate using CellASIC ONIX2 microfluidic system (Merck Millipore) according to the manufacturer's instructions in an anaerobic workstation. TPGY medium was perfused into the microfluidic chamber with the pressure of 13.8 kPa for 6 h to enable sufficient spore germination. After 20 µM of CBO1751 or control DB was perfused into the microfluidic plate to replace TPGY medium, phase-contrast images of newly germinated cells were taken every 20 s over 60 min using a Leica DMi8 inverted microscope with a 100-fold oil‐immersion lens (Leica Microsystems, Wetzlar, Germany). The images were processed using Metamorph (Universal Imaging, Bedford Hills, NY, USA).

## Supplementary information

Supplementary Information 1.

Supplementary Information 2.

## Data Availability

All data are available from the corresponding authors upon reasonable request.
