# Supplementary material for: Phage lysin that specifically eliminates Clostridium botulinum Group I cells
Source: Sci Rep. 2020 Dec 9;10:21571. doi: 10.1038/s41598-020-78622-6 (PMC7725837; doi:10.1038/s41598-020-78622-6)
Supplement: Supplementary file 1 — Supplementary Information 1. [file 41598_2020_78622_MOESM1_ESM.docx]

**Supplementary information**

**Phage lysin that specifically eliminates *Clostridium botulinum* Group I cells**

Zhen Zhang, Meeri Lahti, François P. Douillard, Hannu Korkeala, Miia Lindström*

Department of Food Hygiene and Environmental Health, Faculty of Veterinary Medicine, University of Helsinki, Helsinki, Finland

*Corresponding author:

Miia Lindström

Department of Food Hygiene and Environmental Health

Faculty of Veterinary Medicine

University of Helsinki

P. O. Box 66, 00014 Helsinki, Finland

[miia.lindstrom@helsinki.fi](mailto:miia.lindstrom@helsinki.fi)

| **Table S1.** Highly conserved CBO1751 homologs in *Clostridium botulinum* Group I and *Clostridium sporogenes* based on BLAST analysis. | | |
| --- | --- | --- |
| **Strains** | **GenBank accession number/NCBI reference sequence** | **Identity against CBO1751 (BLASTp, ≥98% coverage)** |
| ***Clostridium botulinum* Group I** |  |  |
| ATCC 25763, Prevot 910, Prevot Dewping | KOR52872 | 98 % |
| Ba4 657, CDC69043, CFSAN034200 | ACQ54548 | 96 % |
| CDC66221 | WP_045905676 | 92 % |
| A2 Kyoto, Af650, 429-13, H074240406, H074240407, H093320637, H093440236, H093440416, H093520024, H093520263, H093660253, H093660254, H093760187, H094020617, H094160823, H094240742, H094460264, H094500386, H094780541, H094860240, H094920429, H094940215, H095040074, H095200066, H111860974c, H111860974g, H111860975g, H111880801, H111880801g, H112060329, H112100518, H112480657, IFR 12/040, IFR 18/031, IFR 18/032, IFR 18/035, IFR 18/036, IFR 18/037, IFR 18/039, IFR 18/044, SU0801, SU0807, SU0972, SU0994, SU0998, SU1054, SU1064, SU1072, SU1074, SU1112, SU1259, SU1274, SU1275, SU1887, SU1891, SU1917, SU1934, SU1937 | ACO85132 | 92 % |
| F160 | APQ95575 | 91 % |
| H091280045, H091420008, H091140481, H091280046 | NFD29677 | 91 % |
| H102120681, H102120680 | NFB57057 | 91 % |
| 429-13 | OPD21397 | 91 % |
| A2 117 | KEI79078 | 91 % |
| Prevot 1542, IFR 18/053, 115B, Colworth BL262, 12885A, NCTC 3815 | OSB01544 | 90 % |
| CDC54088 | WP_024933164 | 90 % |
| AM1195, Mfbjulcb3, AM370 | AUM96313 | 90 % |
| Af84, CDC54091, SU1304, SU1306, 2G, IFR 16/385, IFR 18/081 | EPS55316 | 90 % |
| CDC_67086 | RUT55501 | 90 % |
| CDC_54064, 1169, SU1169, SU0945 | WP_061333624 | 90 % |
| A2 Kyoto, Af650, 429-13, F1413/94, H074240406, H074240407, H093320637, H093440236, H093440416, H093520024, H093520263, H093660253, H093660254, H093760187, H094020617, H094160823, H094240742, H094460264, H094500386, H094780541, H094860240, H094920429, H094940215, H095040074, H095200066, H111860974c, H111860974g, H111860975g, H111880801, H111880801g, H112060329, H112100518, H112480657, IFR 12/040, IFR 18/031, IFR 18/032, IFR 18/035, IFR 18/036, IFR 18/037, IFR 18/039, IFR 18/044, SU0801, SU0807, SU0994, SU0998, SU1054, SU1064, SU1072, SU1074, SU1259, SU1274, SU1275, SU1887, SU1891, SU1917, SU1934, SU1937 | ACO85053 | 89 % |
| H125100367 | NFB30016 | 89 % |
| CDC 7827 | NFO71966 | 89 % |
| A2B3 87 | KEI96546 | 89 % |
| CFSAN064329 | AWB30064 | 89 % |
| Ba4 657, H142660711 | ACQ54391 | 89 % |
| DFPST0029 | AWB17271 | 89 % |
| CFSAN024410 | KGO13382 | 89 % |
| CDC 1656 | OSB03164 | 89 % |
| CDC66008 | RHW65359 | 89 % |
| BrDura, BrDuraAf | AUN19030 | 88 % |
| IFR 18/002 | NFM45169 | 88 % |
| CDC 795 | WP_061310873 | 88 % |
| A3 Loch Maree, NCTC 2012, Colworth BL30, IFR 18/110 | ACA55839 | 87 % |
| B305, SU0305 | AUN11554 | 87 % |
| H074240407, H074240406 | NFC74729 | 87 % |
|  |  |  |
| Table S1 continued. |  |  |
| **Strains** | **GenBank accession number/NCBI reference sequence** | **Identity against CBO1751 (BLASTp, ≥98% coverage)** |
| ***Clostridium botulinum* Group I** |  |  |
| 1141-11 | OPD29617 | 87 % |
| F Langeland, F 230613, Walls 8G, FT 14, FT 15, IFR 18/146, 5052 | ABS42254 | 87 % |
| Bf, CDC 297, CDC 1436, CDC69057, H130580885, H134990001, F2534/89 | EDT86373 | 87 % |
| CDC_69096 | APU60718 | 87 % |
| A2 Kyoto, Af650, F1413/94, H094460264, H094860240, H094920429, H095200066, H112480657, IFR 12/040, SU0801, SU0807, SU0994, SU1054, SU1064, SU1072, SU1074, SU1259, SU1274, SU1275, SU1891, SU1917 | ACO86185 | 87 % |
| A634, CDC54085, SU0634, SU0801, SU0807, SU0945, SU0994, SU1074 | AUN04008 | 86 % |
| AM1195, Mfbjulcb3, AM370 | AUM95325 | 86 % |
| ATCC3502, NCTC 13319, IFR 18/091, 39 KA | CAL83867 | 86 % |
| CFSAN002367, ATCC 17862, AM1295, B515, B609, KF Meyer 33, McClung 844, SU0635W, VPI 7124, 33A | EPS48428 | 86 % |
| H111880801, H112100518, H111860974c, H111860974g, H112060329, H111880801g | NFA22290 | 86 % |
| A2 Kyoto | ACO85146 | 85 % |
| CDC_1632, CDC_67071, CDC68016 | APH15108 | 85 % |
| AIP 580-86 | NFL55696 | 83 % |
| SU0998, H094780541, H095040074, H094240742, H093660254, H094020617, H094500386, H094160823, H093760187, H094940215, H093520263, H093660253, H093440416, H093320637, H093440236, H093520024 | NFC06755 | 83 % |
| ***Clostridium sporogenes*** |  |  |
| CDC24533 | WP_045905020 | 93 % |
| PA 3679, IFR 18/152, IFR 18/150, IFR 18/153, IFR 18/151, IFR 18/071, IFR 18/092, IFR 18/080, IFR 18/099 | OQP96336 | 92 % |
| CLS_DGF_0088_06 | OSB16679 | 91 % |
| IFR 18/067 | NFP92425 | 90 % |
| IFR 18/024 | NFH40140 | 90 % |
| CDC23284 | WP_045887044 | 90 % |
| IFR 18/087 | NFQ85400 | 90 % |
| IFR 18/028, IFR 18/023, IFR 18/145, IFR 18/144, IFR 18/072 | NFG96870 | 90 % |
| IFR 18/070, IFR 18/102, IFR 18/101, IFR 18/100, IFR 18/098, IFR 18/069 | NFQ59290 | 89 % |
| IFR 18/061, IFR 18/062 | NFQ01083 | 89 % |
| Colworth BL177, IFR 18/041, IFR 18/040, IFR 18/034, IFR 18/138, IFR 18/109, IFR 18/103, IFR 18/073, IFR 18/064, IFR 18/063, IFR 18/058 | NFF99411 | 89 % |
| IFR 18/122 | NFT27081 | 87 % |
| 87-0535, Colworth BL179, IFR 18/077 | WP_045518633 | 87 % |
| IFR 18/087 | NFQ85107 | 86 % |
| UC9000 | KOY66370 | 85 % |
| IFR 18/023, IFR 18/028, IFR 18/145, IFR 18/144, IFR 18/072 | NFH30989 | 84 % |
| ***Clostridium botulinum* Group I*/Clostridium sporogenes*** |  |  |
| *C. botulinum* NCTC13037, *C. sporogenes* PA 3679 isolate 2007, ATCC 7955 NCA3679 isolate 1990, ATCC 7955 NCA3679 isolate 2007, IFR 18/061, IFR 18/062 | STC76931 | 89 % |
| *C. botulinum* R1125/03, R1135/03, *C. sporogenes* 2113/01, IFR 18/149, FT236, 1779, FT236 | NFA60807 | 86 % |
| *C. botulinum* R1135/03, *C. sporogenes* 2113/01, IFR 18/149, FT236, 1779 | NFV68554 | 86 % |

**Table S2.** Bacterial strains and plasmids.

| Bacterial strain or plasmid | Relevant characteristics | Reference or source^a^ |
| --- | --- | --- |
| *Clostridium botulinum* |  |  |
| ATCC3502 | Group I, produces type A botulinum neurotoxin (BoNT/A) | (1) |
| NCTC2916 | Group I, produces BoNT/A, carries a silent gene for BoNT/B | (2) |
| 62A | Group I, produces BoNT/A | (2) |
| ATCC19397 | Group I, produces BoNT/A | (3) |
| 133-4803 | Group I, produces BoNT/B | (4) |
| 213B | Group I, produces BoNT/B | (4) |
| F Langeland | Group I, produces BoNT/F | (2) |
| Eklund 2B | Group II, produces BoNT/B | (2) |
| CB11/1-1 | Group II, produces BoNT/E | (5) |
| K126 | Group II, produces BoNT/E | (5) |
| Eklund 202F | Group II, produces BoNT/F | (2) |
| BKT2873 | Group III, produces BoNT/CD | (6) |
| 16868 | Group III, produces BoNT/D | (6) |
| *Clostridium* spp. |  |  |
| *Clostridium sporogenes* NINF45 | Nontoxigenic, closely related to *C. botulinum* Group I | (7) |
| *Clostridium baratii* CCUG24033 | Nontoxigenic, Also called *C. botulinum* Group V, type strain | (7) |
| *Clostridium butyricum* BL86/13 | Also called *C. botulinum* Group VI, produces BoNT/E | DFHEH/QI |
| *Clostridium perfringens* ATCC13124 | Gas gangrene isolate, produces α-toxin | (8) |
| *Clostridium difficile* CD-UN5/11-14 | Clinical isolate, causes colitis | DFHEH |
| Other species |  |  |
| *Bacillus cereus* ATCC14579 | *B. cereus* type strain | ATCC |
| *Bacillus subtilis* 1012M15 | Derivative strain from *B. subtilis* 168 | DFHEH |
| *Escherichia coli* Rosetta 2(DE3) pLysS | F^–^ *ompT hsdS*_B_(r_B_^–^ m_B_^–^) *gal dcm* (DE3) pLysSpRARE2^3^ (Cam^R^) | Merck Millipore |
| Plasmids |  |  |
| pET 21b | P_T7_, Kan^R^, *ori* pBR322, *ori* f1, *lac*I, N-terminal T7 Tag, C-terminal 6xHis tag | Merck Millipore |
| pET 21b::*cbo1751* | pET 21b carrying *cbo1751* that is cloned in the NheI/SalI restriction sites | This study |
| Rosetta 2(DE3) pLysS-pET 21b::*cbo1751* | *E. coli* Rosetta 2(DE3) pLysS carrying plasmid pET 21b::*cbo1751* | This study |

**^a^**DFHEH, Department of Food Hygiene and Environmental Health, Faculty of Veterinary Medicine, University of Helsinki, Finland. QI, Quadram Institute, United Kingdom. ATCC, American Type Culture Collection. References: (1) Zhang Z, Korkeala H, Dahlsten E, Sahala E, Heap JT, Minton NP, Lindström M. 2013. PLoS Pathog. 9:e1003252; (2) Dahlsten E, Korkeala H, Somervuo P, Lindström M. 2008. Int J Food Microbiol. 124:108-111; (3) Ihekwaba AE, Mura I, Peck MW, Barker GC. 2015. Pathog Dis. 73:ftv084; (4) Nevas M, Lindström M, Hielm S, Björkroth KJ, Peck MW, Korkeala H. 2005. Appl Environ Microbiol. 71:1311-1317; (5) Zhang Z, Hintsa H, Chen Y, Korkeala H, Lindström M. 2013. Appl Environ Microbiol. 79:3856-3859; (6) Skarin H, Segerman B. 2014. PLoS One. 9:e107777; (7) Keto-Timonen R, Heikinheimo A, Eerola E, Korkeala H. 2006. J Clin Microbiol. 44:4057–4065; (8) Lahti P, Lindström M, Somervuo P, Heikinheimo A, Korkeala H. 2012. PLoS One. 7:e46162.

**Figure S1.** Amino acid sequence alignment of CBO1751 with previously described clostridial phage lysins.

CLUSTAL 2.1 Multiple Sequence Alignments

Sequence 1: CBO1751 253 aa

Sequence 2: *C.tyrobutyricum*_CTP1L 274 aa

Sequence 3: *C.difficile*_CD27L 270 aa

Sequence 4: *C.difficile*_PlyCD 262 aa

Sequence 5: *C.perfringens*_Ply3626_WP_003459500.1 347 aa

Sequence 6: *C.perfringens*_PlyCpAmi_YP_696189.1 337 aa

Sequence 7: *C.perfringens*_PlyCP39O_ACE82009.1 213 aa

Sequence 8: *C.perfringens*_PlyCP26F_AEA86246.1 212 aa

Sequence 9: *C.perfringens*_PlyCM_ABG83309.1 342 aa

Sequence 10: *C.perfringens*_CP25L_AGH27916.1 377 aa

Sequence 11: *C.perfringens*_Psm_phiSM101_YP_699978.1 342 aa

Sequence 12: *C.perfringens*_ZP173_AXP19870.1 335 aa

Sequence 13: *C.perfringens*_ZP278_AXP19871.1 351 aa

Sequence 14: *C.perfringens*_LysCPS2_AWG96523.1 226 aa

Sequences (1:2) Aligned. Score: 9

Sequences (1:3) Aligned. Score: 22

Sequences (1:4) Aligned. Score: 22

Sequences (1:5) Aligned. Score: 23

Sequences (1:6) Aligned. Score: 27

Sequences (1:7) Aligned. Score: 17

Sequences (1:8) Aligned. Score: 16

Sequences (1:9) Aligned. Score: 11

Sequences (1:10) Aligned. Score: 13

Sequences (1:11) Aligned. Score: 11

Sequences (1:12) Aligned. Score: 7

Sequences (1:13) Aligned. Score: 11

Sequences (1:14) Aligned. Score: 12

*C.difficile*_CD27L -------------MKICITVGHSILKSGACTSADGVVNEYQYNKSLAPVL 37

*C.difficile*_PlyCD -------------LKVVIIPGHTLIGKG--TGAVGYINESKETRILNDLI 35

*C.perfringens*_Ply3626_WP_00345 -------------MKIAERGGHNFQATG----AVGLINETVEDRKVLAAA 33

*C.perfringens*_PlyCpAmi_YP_6961 -------------MKIAVRGGHNFKAKG----ALGIIDETIENRKVYKAL 33

*C.perfringens*_PlyCP39O_ACE8200 -------------MKIALRGGHSPNCKG----ANVLRDEQSCMWALADEV 33

*C.perfringens*_PlyCP26F_AEA8624 -------------MIIGSRYGHSENCRG----AKGLRDEVDAMKPLHFEF 33

*C.perfringens*_PlyCM_ABG83309.1 MQSRNNNNLKGIDVSNWKGNINFQSVKNDGVEVVYIKATEGNYFKDKYAK 50

*C.perfringens*_Psm_phiSM101_YP_ MQSRNNNNLKGIDVSNWKGNINFESVKNDGVEVVYIKATEGNYFKDKYAK 50

*C.perfringens*_ZP173_AXP19870.1 -------MLKGIDVSEHQGRIDWERVKGN-IDFAILRAGYGRNNIDKQFI 42

*C.perfringens*_CP25L_AGH27916.1 -------------MYINQSNIKFNGLRYG-NDPNKIIIHNADATSCSVYD 36

*C.perfringens*_LysCPS2_AWG96523 -------------MKIIQSNIHFNGNKAGGNNPKEIIVHHSEHSTANVYD 37

CBO1751 -------------MKIGIDCGHTMSGADY--GAVGIKAESNLTREVGTKV 35

*C.perfringens*_ZP278_AXP19871.1 MQSRSDSNFKGIDISNWQKGINLNQLKERGYDVCYIKITEGKGYVDPCFE 50

*C.tyrobutyricum*_CTP1L -------MKKIADISNLNGNVDVKLLFNLGYIGIIAKASEGGTFVDKYYK 43

: .

*C.difficile*_CD27L ADTFRKE----GHKVDVIICPEKQFKTKNEEKSYKIPRVNSGGYDLLIEL 83

*C.difficile*_PlyCD VKWLKIG----GATVYTGRVDE---SSNHLADQCAIANKQETDLAVQIHF 78

*C.perfringens*_Ply3626_WP_00345 YKYTKAA----GYDVLDVTPGNC---DSNTDLILGVNKAERFGAELFLSY 76

*C.perfringens*_PlyCpAmi_YP_6961 IKYLNIA----GHNVIDVTPGEC---DINTDLYLGVQKAKENNSELFLSI 76

*C.perfringens*_PlyCP39O_ACE8200 EKVLTSH----GHTVVRCETTLS---NEREDVRQGAKKG--YNCDMFISL 74

*C.perfringens*_PlyCP26F_AEA8624 KKIMEQY----GHTIIDCCSNAN---TQNGELSEGARKANAQILDLFISW 76

*C.perfringens*_PlyCM_ABG83309.1 QNYERAKEQGLRVGFYHFFRAN--KGAKDQANFFVNYLNEIGAVNYDCKL 98

*C.perfringens*_Psm_phiSM101_YP_ QNYEGAKEQGLSVGFYHFFRAN--KGAKDQANFFIDYLNEIGAVNYDCKL 98

*C.perfringens*_ZP173_AXP19870.1 RNIEECNRLCIPVGIYWFSYAWNEEMAKNEARYVLEAIKGY-RVDYPISY 91

*C.perfringens*_CP25L_AGH27916.1 IDRWHKGNGWSGIGYDYFIRKEGSVWTGRPENAIGAHTIGQNSSSIGICL 86

*C.perfringens*_LysCPS2_AWG96523 IDRWHKDKGWCGIGYHYFIDKQGNIYTGRPEDWTGAHCIDHNTKSIGICL 87

CBO1751 ISKLQVL----GHTVIKCYKDTCS--SLNDSLSYRTNTANNNNVDLYVSI 79

*C.perfringens*_ZP278_AXP19871.1 ENYNKAIAAGMKVGVYHYWRGTS---SAIEQANNIVRTLGNKHIDCKIAI 97

*C.tyrobutyricum*_CTP1L QNYTNTKAQGKITGAYHFANFSTIAKAQQEANFFLNCIAGTTPDFVVLDL 93

.

*C.difficile*_CD27L HLNASN---GQGKGSEVLYYSNKGL---EYATRICDKLGT--VFKNRGAK 125

*C.difficile*_PlyCD NSNATT---STPVGTETIYKTNNGK---TYAERVNTRLAT--VFKDRGAK 120

*C.perfringens*_Ply3626_WP_00345 HFDKCYDEYNGALGVACWICATGGK-AEEYAKSIVDTIAAGTGLKNRGVK 125

*C.perfringens*_PlyCpAmi_YP_6961 HFDKAYDKYEGPLGTGTWIYGRGGK-AEIYAKRIVDNLSKGTGLKNRGVK 125

*C.perfringens*_PlyCP39O_ACE8200 HMNASD---GRGNGTEAWVARSARSSIKEIASRLCKNYAT-LGLQNRGVK 120

*C.perfringens*_PlyCP26F_AEA8624 HGNK-----GGGQGCEAWIANNSR--AKPYAERMCKNFSS-LGFKNRGVK 118

*C.perfringens*_PlyCM_ABG83309.1 ALDIETT-------EGVGARDLTSMCIEFLEEVKRITGKEVVVYTYTSFA 141

*C.perfringens*_Psm_phiSM101_YP_ ALDIETT-------EGVGVRDLTSMCIEFLEEVKRLTGKEVVVYTYTSFA 141

*C.perfringens*_ZP173_AXP19870.1 DLEYDTLNYASKNGVTIGKRLATDMVKAFCDEINR-NGYRAMNYTNQDFL 140

*C.perfringens*_CP25L_AGH27916.1 EGAFMRE---------KPTRAQLNSLYELIADIRA-RRGNLPVYGHKDFN 126

*C.perfringens*_LysCPS2_AWG96523 QGRLQVE---------SVTDAQYNALLWLIKDIRN-RRGNMPIYGHKELN 127

CBO1751 HFNCYNG---SAYGAEVFTYGGKS----FTEASRVLNNICALGYTNRGIK 122

*C.perfringens*_ZP278_AXP19871.1 DVEQTDG---------LSYGELNNSVLQLAEELERLIGAEVCIYCNTNYA 138

*C.tyrobutyricum*_CTP1L EQQCTG--------------DITDACLAFLNIVAK--KFKCVVYCNSSFI 127

*C.difficile*_CD27L LDKR-LYILNSSKPTAVLIESFFCDNKEDYDKAKKLGHEGIAKLIVEG-- 172

*C.difficile*_PlyCD SDVRGLYWLNHTIAPAILIEVCFVDSKADTDYYVNN-KDKVAKLIAEG-- 167

*C.perfringens*_Ply3626_WP_00345 VNPK-LYELRKTSMPAVIVEVCFCEATEDVRIYKEKGADLIGKLIAEGVC 174

*C.perfringens*_PlyCpAmi_YP_6961 ENSK-LYELRKTSMPAVLVEVCFCEATEDVRIYREKGPDLIGKLIAEAIN 174

*C.perfringens*_PlyCP39O_ACE8200 -EKN-YWEMTDTNCPNIIFETMFCDDKHDIDIWASTSWDKLARLIAN--- 165

*C.perfringens*_PlyCP26F_AEA8624 YSDK-YYEMRNINAPNIIFETLFLDSEKDISIWSPIPYEVMARYLAN--- 164

*C.perfringens*_PlyCM_ABG83309.1 NNNLDS-RLSSYPVWIAHYGVNTPGANNIWSEWVGFQYSENGSVAGVSGG 190

*C.perfringens*_Psm_phiSM101_YP_ NNNLDS-RLGNYPVWIAHYGVNTPGANNIWSSWVGFQYSENGSVAGVNGG 190

*C.perfringens*_ZP173_AXP19870.1 LNKFYMNELTNYPLWYAWYNSKLNRDCAIW------QYSENGQVPGIPGS 184

*C.perfringens*_CP25L_AGH27916.1 NTDCPGINFPLEQFKNNSYRPTGGEIVSDNGFYRSDEERTNATIVGEGNI 176

*C.perfringens*_LysCPS2_AWG96523 STDCPG-NLDLNKLRTD----VNNKVVDSNGGYT-----ENATVVN---- 163

CBO1751 DGSN-LYVLKHTKAKAMLIECCFCDNAGDMNRYN---AENMANAIVKG-- 166

*C.perfringens*_ZP278_AXP19871.1 RNVLDS-RLGKYSLWVAHYGVNKPGDNPIWDKWAGFQYSENGTSNVNGSL 187

*C.tyrobutyricum*_CTP1L KEHLNS-KICAYPLWIANYGVATP-AFTLWTKYAMWQFTEKGQVSGISG- 174

: .

*C.difficile*_CD27L ----------------------------------------------VLNK 176

*C.difficile*_PlyCD ----------------------------------------------ILNK 171

*C.perfringens*_Ply3626_WP_00345 KVAGGQVP-------------------------------------GTVIE 187

*C.perfringens*_PlyCpAmi_YP_6961 EKE--------------------------------------------IEE 180

*C.perfringens*_PlyCP39O_ACE8200 ----------------------------------------------AIDP 169

*C.perfringens*_PlyCP26F_AEA8624 ----------------------------------------------AIDP 168

*C.perfringens*_PlyCM_ABG83309.1 -----------------------------------------------CDM 193

*C.perfringens*_Psm_phiSM101_YP_ -----------------------------------------------CDM 193

*C.perfringens*_ZP173_AXP19870.1 S----------------------------------------------VDM 188

*C.perfringens*_CP25L_AGH27916.1 EVLDKNCKVIENRYISSLDRVFVLGIYPASKYIEIIYPAGNEKYHAYISI 226

*C.perfringens*_LysCPS2_AWG96523 --------------------------------------------------

CBO1751 ----------------------------------------------LVGQ 170

*C.perfringens*_ZP278_AXP19871.1 D----------------------------------------------LDE 191

*C.tyrobutyricum*_CTP1L ----------------------------------------------YIDF 178

*C.difficile*_CD27L NINNE---GVKQMYKH---------------------------------- 189

*C.difficile*_PlyCD SISNSQGGGENKVYEN---------------------------------- 187

*C.perfringens*_Ply3626_WP_00345 NVEYEVQESKPVPVYDRNKFKTNARALVNLDPRDRASGIYEDLGEIYKDE 237

*C.perfringens*_PlyCpAmi_YP_6961 NIKPEGQED---SLKEKFLKSTNAKAIANLDPRDNPSSIYKDLGEIYKGE 227

*C.perfringens*_PlyCP39O_ACE8200 NIPLEKEQD----------------------------------------- 178

*C.perfringens*_PlyCP26F_AEA8624 NIPLEKEQD----------------------------------------- 177

*C.perfringens*_PlyCM_ABG83309.1 NEFTNGIFIDSNNFTLDNATTKNVSIKLNIRAKGTTNSKVIGSIPANEKF 243

*C.perfringens*_Psm_phiSM101_YP_ NEFTEEIFIDSSNFNLDNATTKNVSTKLNIRAKGTTNSKIIGSIPAGETF 243

*C.perfringens*_ZP173_AXP19870.1 NYCYEDFLK--KDFTLENATTCNVDTELNIRAKGTTGATIVGSIPAGDRF 236

*C.perfringens*_CP25L_AGH27916.1 ENYSRISFDYHMQYKNDNGVTYVWWDSEDVNVKEHNEELQANQKASPMYR 276

*C.perfringens*_LysCPS2_AWG96523 --------------------------------------------------

CBO1751 TTSSTPSKPTDNNNNS---------------------------------- 186

*C.perfringens*_ZP278_AXP19871.1 FTEEIFINKESSKVTENKLFSTNARALVALDPRDNPSDNYNDLGEIYEGE 241

*C.tyrobutyricum*_CTP1L SYITDEFIKYIKGEDEVEN------------------------------- 197

*C.difficile*_CD27L ----------------TIVYDGEVDKISATVVGWGYNDGK---------- 213

*C.difficile*_PlyCD ----------------VIVYTGDADKVAAQILHWQLKDS----------- 210

*C.perfringens*_Ply3626_WP_00345 RFYVLPEVCDKGDYLPVLYWKDGANRASNKVWVSSKQKYMMIDTYHRVVN 287

*C.perfringens*_PlyCpAmi_YP_6961 RIRVLPEICDKKDYLPIIYWKDTTNIESQKVWVSAKQNYLKIDTNATVIN 277

*C.perfringens*_PlyCP39O_ACE8200 ------------------YYRVCVQRFTN--------------------- 189

*C.perfringens*_PlyCP26F_AEA8624 ------------------YYRVCVQRFTN--------------------- 188

*C.perfringens*_PlyCM_ABG83309.1 KIKWVDEDYLGWYYVEYNGIVGYVNADYVEKLQMATTHN----------- 282

*C.perfringens*_Psm_phiSM101_YP_ KIKWVDEDYLGWYYVEYNGVVGYVNADYVEKLQMATTYN----------- 282

*C.perfringens*_ZP173_AXP19870.1 RIKWVDSDYLGWYYIEYQGITGYVSQDYVEKLQMATTCN----------- 275

*C.perfringens*_CP25L_AGH27916.1 VGKWLRVTFYRTDGTPSDGFVRYEGEQAVKFYEEEKIKEG-------IVK 319

*C.perfringens*_LysCPS2_AWG96523 --------------------------------------------------

CBO1751 ----------------WINLDGKTGTICTP-------------------- 200

*C.perfringens*_ZP278_AXP19871.1 RIQVLAEVCDKEDYLPVKYWKDSEGRESGKVWIRSKQDYMMIDTYHRVFN 291

*C.tyrobutyricum*_CTP1L ----------------LVVYNDGADQRAAEYLADRLACP----------- 220

*C.difficile*_CD27L -ILICDIKDYVPGQTQNL-YVVGGGACEKISSITKEKFIMIKGNDRFDTL 261

*C.difficile*_PlyCD --LIIEASSYKQGLGKKV-YVVGGEANKLVKGD-----VVINGADRYETV 252

*C.perfringens*_Ply3626_WP_00345 VVTELDARYEPSPNSNRMGYVCNAERVYVHKIEGNYALCTYFAGEGYKTA 337

*C.perfringens*_PlyCpAmi_YP_6961 VVTELDARYIKSQRSSKMGWVKNGERLYVHKIESGYALGTYFASNGYKTA 327

*C.perfringens*_PlyCP39O_ACE8200 ---KEDAEKAQQRISNELGYYCFAEKI----------------------- 213

*C.perfringens*_PlyCP26F_AEA8624 ---KEDAEKAQQRISNELGYYCFAEKI----------------------- 212

*C.perfringens*_PlyCM_ABG83309.1 VSTFLNVREEGSLNSRIVDKINTGDIFRIDWVDSDFIGWYRVTTKNGKVG 332

*C.perfringens*_Psm_phiSM101_YP_ VSTFLNVREEGSLNSRIVDKINSGDIFRIDWVDSDFIGWYRITTKNGKVG 332

*C.perfringens*_ZP173_AXP19870.1 VDSVLNVRAEGNTSSNIVATINPGEVFRIDWVDSDFIGWYRITTANGANG 325

*C.perfringens*_CP25L_AGH27916.1 VNTYLNVRD--SINGNIIGKVFNGEEVSIIWTKD---GWYYIDYNTNHGK 364

*C.perfringens*_LysCPS2_AWG96523 VNSYLNVRS--KPSDEIIGKLFPNERIQVNWVDSNYLGWYYITYRVNETN 211

CBO1751 --SGVNIREKKSTSSRILGALPNGAKVQLYRKEGDWIHIYYPPHG----- 243

*C.perfringens*_ZP278_AXP19871.1 VITQLDARYEPSSDSATMGYVKNGERLYVHRTEGNYSLCTYFAGNGYKTA 341

*C.tyrobutyricum*_CTP1L --TINNARKFDYSNVKNVYAVGGNKEQYTSYLTTLIAGSTRYTTMQAVLD 268

: :

*C.difficile*_CD27L -----YKALDFINR-- 270

*C.difficile*_PlyCD -----KLALQEIDKL- 262

*C.perfringens*_Ply3626_WP_00345 -----WFTAKYLERI- 347

*C.perfringens*_PlyCpAmi_YP_6961 -----WFTAKYISLD- 337

*C.perfringens*_PlyCP39O_ACE8200 ----------------

*C.perfringens*_PlyCP26F_AEA8624 ----------------

*C.perfringens*_PlyCM_ABG83309.1 -----FVNAEFVKKL- 342

*C.perfringens*_Psm_phiSM101_YP_ -----FVNAEFVKKL- 342

*C.perfringens*_ZP173_AXP19870.1 -----FVKSDFVKKL- 335

*C.perfringens*_CP25L_AGH27916.1 --KRGYVSSKYVEEV- 377

*C.perfringens*_LysCPS2_AWG96523 KLKSGYVSAKYIKKD- 226

CBO1751 ----GYVYEKYIRY-- 253

*C.perfringens*_ZP278_AXP19871.1 -----WFTAKYLERI- 351

*C.tyrobutyricum*_CTP1L ----------YIKNLK 274

**Figure S2.** SDS-PAGE analysis of the purification of CBO1751. M, Precision Plus Protein Dual Color Standards (Bio-Rad, Hercules, USA). S, soluble fraction of bacterial lysate. I, insoluble fraction of bacterial lysate. B, flow-through fraction of binding buffer. W, flow-through fraction of wash buffer. E1-E5, eluted fractions of CBO1751.


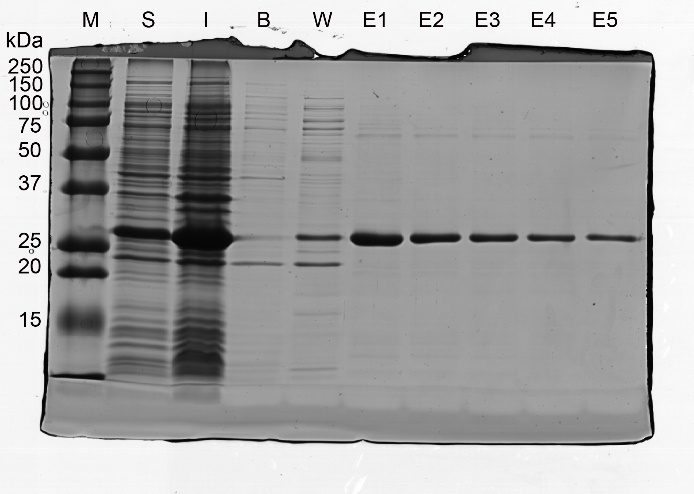


**Figure S3.** Representative microscopic time lapse (min:sec) images of newly germinated cells of *Clostridium botulinum* ATCC3502 after control treatment with dialysis buffer (DB). Scale bars, 2 μm. Square-shape dots and platform are intrinsic parts of the microfluidic chamber.


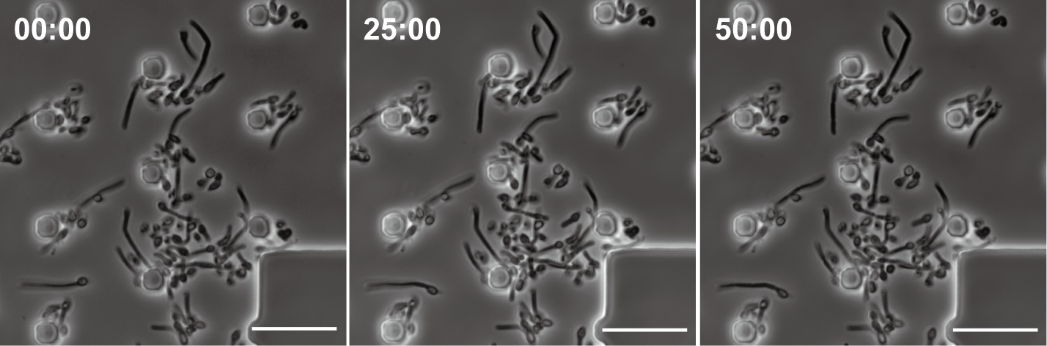


**Supplementary Video Legend**

**Video S1.** Representative time-lapse (min:sec) movie of *Clostridium botulinum* ATCC3502 cell lysis after treatment with 20 μM of CBO1751. Scale bars, 2 μm. Square-shape dots are intrinsic pillars of the microfluidic chamber.
